# Does cardiac development provide heart research with novel therapeutic approaches?

**DOI:** 10.12688/f1000research.15609.1

**Published:** 2018-11-06

**Authors:** Angeliqua Sayed, Mariana Valente, David Sassoon

**Affiliations:** 1 Cellular, Molecular, and Physiological Mechanisms of Heart Failure, Paris-Cardiovascular Research Center (PARCC), European Georges Pompidou Hospital (HEGP), INSERM U970, F-75737 Paris Cedex 15, Paris, France

**Keywords:** hypoxia, glycolytic, oxidative, cardiac progenitor, epicardium

## Abstract

Embryonic heart progenitors arise at specific spatiotemporal periods that contribute to the formation of distinct cardiac structures. In mammals, the embryonic and fetal heart is hypoxic by comparison to the adult heart. In parallel, the cellular metabolism of the cardiac tissue, including progenitors, undergoes a glycolytic to oxidative switch that contributes to cardiac maturation. While oxidative metabolism is energy efficient, the glycolytic-hypoxic state may serve to maintain cardiac progenitor potential. Consistent with this proposal, the adult epicardium has been shown to contain a reservoir of quiescent cardiac progenitors that are activated in response to heart injury and are hypoxic by comparison to adjacent cardiac tissues. In this review, we discuss the development and potential of the adult epicardium and how this knowledge may provide future therapeutic approaches for cardiac repair.

## Introduction

Cardiovascular diseases represent a prevalent medical challenge and are a leading cause of morbidity and mortality in developed societies. Although multiple approaches have been developed in the last two decades to repair or replace damaged myocardium, progress to date has been modest and cardiomyocyte replacement in adult hearts remains elusive. While understanding the loss of cardiac muscle and its eventual replacement is an important goal for cardiac research, there are additional cellular targets that hold promise. These include abrogating scar tissue formation following heart injury or promoting robust revascularization to the injured heart tissue. Here, we present an overview of the different cell types that participate in mammalian heart development as well as how the microenvironment participates in cardiac remodeling. Typically, tissue repair is driven by tissue-resident progenitors. Although significant debate exists regarding the identity and functional potential of adult cardiac progenitor cells (CPCs), there is an emerging consensus that the adult epicardium contains cells with progenitor potential that can participate in cardiac remodeling after injury. Whereas the heart undergoes profound changes during embryonic, fetal, and early postnatal growth, the epicardium maintains many characteristics found during heart development. Consequently, the adult epicardium is an important cellular compartment that may provide solutions to ameliorating heart repair. We focus particularly on rodent models that have allowed the identification of progenitor populations that give rise to the heart as well as for the study of specific cell types in response to injury.

## Cardiac progenitor populations during development

**Figure 1.**
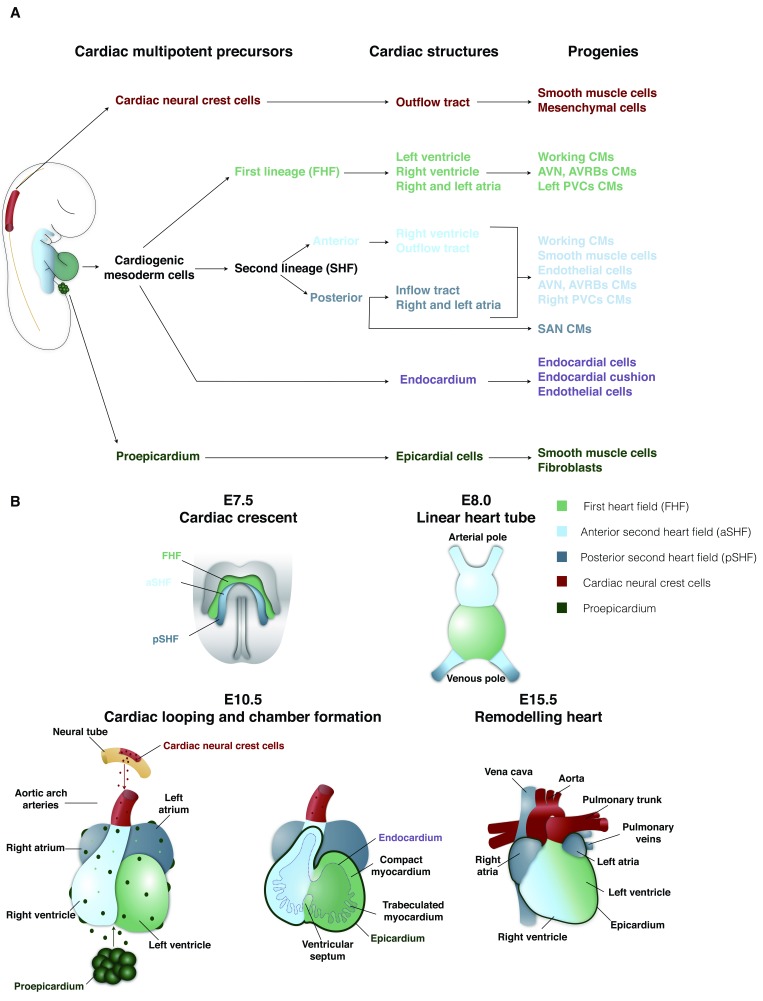
Patterning of mouse heart development. ( **A**) Hierarchical relationship of the different cardiac developmental progenitors and their progeny. ( **B**) Main developmental steps of heart morphogenesis. A color code was assigned and followed to define each cardiac progenitor population and their progeny. AVN, atrioventricular node; AVRB, atrioventricular ring bundle; CM, cardiomyocyte; E, embryonic day; FHF, first heart field; PVC, peripheral ventricular conduction system; SAN, sinoatrial node; SHF, second heart field.

The mature heart consists of an inner layer of endocardial cells (endocardium) and an outer layer of epicardial cells (epicardium) that surround the myocardium. The myocardium is composed of cardiomyocytes as well as cells of the conductive system, smooth muscle, and endothelium and stromal cells/fibroblasts and valvular interstitial cells ^1, 2^. Three sets of multipotent precursors have been identified that give rise to cardiac cells: cardiogenic mesoderm cells ^3, 4^, cardiac neural crest cells (cNCCs) ^5^, and proepicardial organ (PEO) cells ^6, 7^ ( Figure 1A). Cardiogenic mesoderm cells give rise to cardiomyocytes by progressive lineage restriction ^8^ that segregate during the onset of gastrulation ^9^. During murine development, a first lineage appears at embryonic day 7.0 (E7.0), forms the cardiac crescent (first heart field, or FHF) ^10, 11^, and is the primary source of the left ventricular cardiomyocytes and primitive atria ^9, 12^. While the cardiac tube begins to contract and pump blood throughout the embryo ^13, 14^, a second lineage appears at E8.0 that constitutes the second heart field (SHF) and gives rise to the right ventricle, both atria, and the outflow tract (OFT) ^9, 12, 15– 17^. Unlike the FHF, the SHF consists of multipotent cells that give rise to cardiomyocytes as well as endothelial cells ^16, 18– 22^ and the smooth muscle of the OFT region ^20, 23, 24^. The multipotent capacity of the SHF has been assessed by using embryonic stem cells ^20^ or *in vivo* analyses at the population level ^16, 21, 25^; thus, the precise nature and clonal behavior of the SHF progenitors warrant further investigation. At the looping heart tube stage, when both FHF and SHF progenitors are in place, myocardium trabeculation occurs, leading to an increase of the endocardial and myocardial surface area that promotes oxygenation of the tissue ^26, 27^. The base of the trabeculae contains a population of proliferative cells—referred to as the compact layer—that are essential to ventricular wall expansion ^9, 28– 30^. It has been proposed that the epicardium secretes mitogenic signals that drive cardiomyocyte proliferation in the compact zone ^31– 33^ ( Figures 1A, 1B).

Another cellular compartment derived from cardiogenic mesoderm SHF consists of specialized endothelial cells (endocardium) that cover the inner surface of the cardiac tube and participate in different heart morphogenesis processes, which include trabeculation and a part of the coronary tree formation ^16, 34– 36^. These two developmental events are intimately related, as the endocardium is essential to promote trabeculation of the ventricles ^27, 37, 38^ and participates in the exchange of gas and nutrients with the blood circulating in the ventricular chambers ^27, 34, 39, 40^. During septation, ventricular trabeculae initiate compaction, increasing the thickness of the ventricular wall ^27, 41, 42^. Endocardial cells surrounding the trabeculae become entrapped in the compacted myocardium and adopt a capillary-like morphology ^43– 45^. The newly formed endocardial-derived capillaries subsequently connect with the epicardial-derived vascular network. In addition, the endocardium is an important source of mesenchymal cells for endocardial cushion development ^46^ and subsequent valvulogenesis and membranous septation ^47– 49^. During the septation, endocardial cells undergo an endothelial-to-mesenchymal transition (EndoMT) and colonize the subjacent cardiac jelly at the level of the auriculo-ventricular canal at E9.5 and the OFT at later stages ^50– 52^ ( Figures 1A, 1B).

Cardiac neural crest derived from the dorsal neural tube at E9.5 migrates through the pharyngeal arches and gives rise to smooth muscle and mesenchymal cells that participate in the septation of the OFT trunk ^5, 53^ ( Figure 1A, B). 

The PEO is an extra-cardiac transient cauliflower-like structure that appears between E8.5 and E10.5 ^54^ adjacent to the venous pole of the heart tube (splanchnic mesoderm-derived) ^6, 55^. PEO cells attach to the myocardium surface and spread out to constitute to the outer layer of the looping heart tube (that is, epicardium) ^56^. A subset of epicardial cells undergoes epithelial-to-mesenchymal transition (EMT) in the subepicardial region referred to as mesenchymal epicardium-derived cells (EPDCs). A small fraction of EPDCs invades the myocardium to give rise to stromal cells/interstitial fibroblasts and coronary vasculature. The contribution of the PEO to cardiomyocytes and endothelial cells has been proposed but remains less clear ^31, 57– 61^. An infrequent contribution to cardiomyocytes by the epicardium has been reported to be restricted to specific cardiac zones (inter-ventricular septum and parts of the atrial myocardium) ^59, 60, 62^. In addition, it has been shown recently that nascent coronary vasculature forms immediately subjacent to the epicardium (subepicardial zone) and contributes to a large proportion of the coronary arteries, veins, and capillaries in the myocardial compact layer ^61, 63^. Following the formation of the initial vascular plexus, the coronary vessels connect to the aorta ^64^. Irrespective of the origin, the coronary vasculature is essential for the subsequent heart morphogenesis and embryonic viability such that deficient coronary development impairs myocardium compaction and leads to embryonic lethality ^28, 65^ ( Figure 1A, B). The cardiac conduction system is composed of specialized cardiomyocytes that assemble into a complex and heterogeneous structure to make up a central conduction system located in the atria (sinoatrial node [SAN], atrioventricular ring bundles, and the atrioventricular node) and a peripheral conduction system in the ventricles (left and right branches of His bundle, LBB and RBB, and left and right peripheral conductive system or the Purkinje fibers) ^66^. SAN cardiomyocytes derive from the posterior SHF; however, lineage analyses revealed an early lineage segregation from the atrial working cardiomyocyte progenitors ^66, 67^. Retrospective clonal analyses showed that the *nlacz* gene transmitted cells are organized into clusters of clonally related cardiomyocytes, which are composed of a mixture of both working and specialized conductive cardiomyocytes ^68^. These *nlacz* clusters were found in all conductive system compartments, except in the SAN, indicating a common origin between the specialized conductive cardiomyocytes and their neighbor working cardiomyocytes ^66, 68, 69^. Of note, this retrospective clonal analysis does not exclude the contribution of other progenitor populations to the conductive system ^69^. Additionally, other cardiovascular progenitors—that is, PEO (EPDCs) ^70^ and cNCCs ^71^—have been suggested to contribute to the development of the conductive system; however, this participation remains controversial ^66, 69, 72^ ( Figures 1A, 1B).

Our understanding of heart development is that specific progenitors arise at discrete developmental stages originating from different embryonic structures to form the heart. Thus, although the development of a tissue and an understanding of the progenitors involved can shed light on whether CPCs exist in the adult tissue, the heart poses a considerably more complex situation. Indeed, the search for adult CPCs has remained difficult and at times contentious regarding their identity as well as their cell fate potentials. While many tissues are capable of robust regeneration following injury, the mammalian heart shows a limited capacity to repair, suggesting that, if CPCs exist, their capacities are extremely limited. One possible reason for this limited capacity is that the CPC microenvironment of the adult is not permissive and thus the microenvironment of the developing heart warrants further study.

## The developmental cardiac progenitor microenvironment

**Figure 2.**
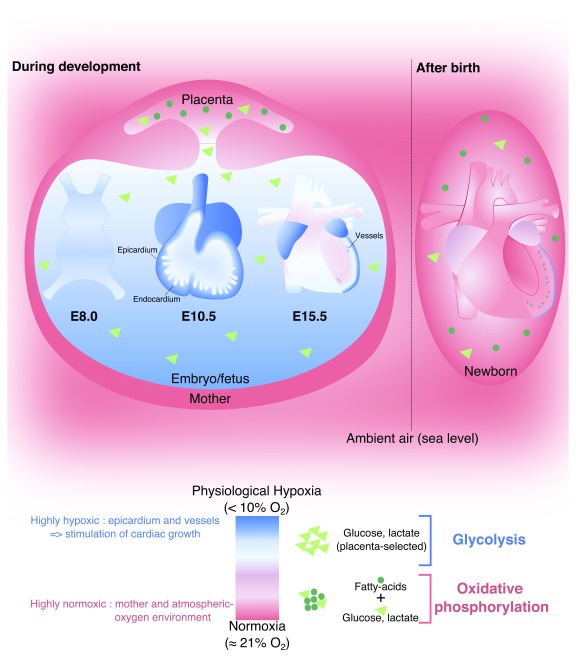
Placenta-selected hypoxic and glycolytic microenvironment and its impact on the heart development and metabolic switch after birth. A color code and symbols were assigned and followed to define the microenvironment condition (depicted in the legend). E, embryonic day.

Placental pregnant females are commonly exposed to normal levels of oxygen (normoxia, 21% oxygen), and in turn the placenta regulates the level of oxygen available during embryonic and fetal development ( Box 1 and Figure 2). The microenvironment of the developing heart is characterized by low levels of oxygen (physiological hypoxia) ( Box 2) ^73^ that promotes glycolytic metabolism ^74, 75^. Oxygen tension is an important signal driving tissue development and maturation ^76– 79^. Several studies have shown that physiological hypoxia regulates multiple cellular processes, including stem cell maintenance, proliferation, and differentiation, particularly in the context of angiogenesis and formation of placenta, heart, cartilage, and bone ^78, 80– 83^. In addition, the developing cardiovascular system is unable to homogeneously deliver nutrients and oxygen throughout the embryonic/fetal heart ^61, 63, 76, 77, 83^. Until mid-gestation, the coronary vasculature is absent and there is a gradient of oxygenation established from the endocardium (more oxygenated and in direct contact with the blood from the chambers) to the epicardium (less oxygenated) ^61, 63, 83^. The myocardial compact layer (epicardial side) is composed of immature cardiomyocytes undergoing higher levels of proliferation under lower oxygen tension as compared with cardiomyocytes in the trabeculae (endocardial side) ^28, 30, 83^. Ultimately, the coronary vasculature is established to meet the increasing demands of the myocardial wall expansion ^44, 61^. It has been demonstrated that hypoxia in combination with vascular endothelial growth factor (VEGF) and platelet-derived growth factor (PDGF) mediates the formation of the primary capillary plexus (angioblasts) as well as subsequent vasculogenesis (formation of new vessels) and angiogenesis (budding and sprouting from pre-existing vessels), leading to the establishment of a functional coronary artery tree ^84– 87^ ( Figure 2).

Box 1. **Placental regulation of fetal oxygen tension**

A complex coordination exists between the fetal cardiovascular system and the placenta to ensure stable oxygen levels ^73, 88– 90^. Low maternal blood oxygen levels, cardiovascular failure, or impairment of placenta formation and function can induce pathological fetal hypoxia ^89– 92^. In pathological hypoxia, the fetus responds by favoring circulation to vital organs such as the brain and heart. In addition, a concomitant activation-specific stress response (for example, Hif-dependent and vascular endothelial growth factor pathways) ^80, 81, 83^ occurs. Chronic fetal pathological hypoxia induces heart septation defects ^93^, myocardial wall thinning, chamber dilation, and epicardium detachment ^79^ as well as decrease of cardiomyocyte proliferation and increase of apoptosis ^94^. Of note, *in utero* stress is also driven by other environmental stressors, including malnutrition that culminates with cardiac malformations and increased predisposition to adult chronic diseases ^95– 98^.

Box 2. **Oxygen balance: normoxic and hypoxic environment**

The ability to maintain oxygen homeostasis is essential for many biological processes, including cell survival and overall development and tissue maintenance. Specialized cells (for example, smooth muscle cells and carotid and neuroepithelial bodies) mobilize proteins and regulate gene expression in order to adapt to hyperoxia or hypoxia ^100– 102^. The environmental oxygen concentration at sea level is 21% ^103^, whereas placental developmental occurs under lower oxygen conditions (2–8%) ^104^. This physiological hypoxia is important for placental and fetal morphogenesis and growth ^73^. During development, fetal arterial blood oxygen tension ranges between 22 and 32 mmHg but in the adult systemic tissues is about 80 to 100 mmHg ^93^. Fetal arterial blood persistently contains less oxygen, suggesting that fetal development occurs in a state of relative hypoxia (that is, physiological hypoxia) in comparison with the adult ^73^.

The murine heart is the first organ to function during development (E8.5) ^13, 14^, and the continuous myocardial contraction requires energy (adenosine triphosphate [ATP]) availability. About 80% of all energy produced by cardiac muscle is consumed by the mechanical activity of the heart, whereas heart morphogenesis relies on the remaining energy ^75^. The developing heart relies primarily on carbohydrates (that is, glucose and lactate) as a source of ATP ^74, 75^. The placenta regulates the availability of metabolic substrates that are delivered to the embryonic/fetal circulation, whereas the fatty acids are blocked ^99^. Glucose is transported to the cytosol of cardiomyocytes and used for glycolysis, glycogen synthesis, or pentosephosphate shunt (metabolic pathway parallel to glycolysis). Glycogen synthesis and storage in fetal cardiomyocytes have been shown to be important sources of phosphorylated glucose, which protects cardiomyocytes from hypoxia. During fetal development, cardiomyocytes have low mitochondrial content ^75, 99^. In summary, embryonic and fetal cardiac development occurs in a physiological hypoxic state, which is essential for the ability of cardiac progenitors to proliferate, self-renew, and differentiate (for example, immature cardiomyocyte hyperplasia or neo-angiogenesis).

## Early postnatal development represents a key transition in cardiac metabolism and complexity

The mammalian heart undergoes a marked increase in workload upon birth, and, in the mouse, the first few weeks of postnatal life are characterized by extensive ventricular remodeling coincident with switch from anaerobic metabolism (glycolysis) to aerobic metabolism (oxidative phosphorylation of fatty acids) ^99, 105^. Experiments performed in rabbits show that while circulating levels of fatty acid are high following birth, the switch from glycolysis to aerobic metabolism occurs only at the end of the first week ^105^ and the neonate heart retains an enriched ability to produce ATP by glycolytic metabolism ^75^ ( Figure 2). Postnatal myocardium growth involves an increase in hemodynamic demands and is characterized by a thickening and vascularization of the ventricular myocardial wall ^27, 106– 108^. Mouse postnatal growth follows three main steps: hyperplasia (postnatal day 0 [P0] to P4), the transitional phase when hyperplasia and hypertrophy processes occur simultaneously (P5 to P15), and hypertrophy (after P15) ^107, 109, 110^. Studies in rodent models have shown that a last round of DNA synthesis and karyokinesis takes place without cytokinesis during the hypertrophy phase, culminating in the bi-nucleation of postnatal cardiomyocytes ^107, 109, 111– 114^. In addition to cardiomyocyte maturation, there is a marked growth and maturation of the coronary vasculature ^115^. The perinatal period is characterized by angiogenesis and an expansion of the capillary network ^115, 116^. This increase of the coronary tree is due to the proliferation of pre-existing capillary endothelial cells and increase of the capillary length ^116^ as well as increases in the thickness, length, and branching of arterial and venous coronary vasculature ^115, 117, 118^.

## Neonatal heart regeneration

In 2011, Porrello and colleagues ^119^ demonstrated that the mouse neonatal heart regenerates in response to ventricular apex resection as well as experimentally induced myocardial infarction (MI) ^120^. The regenerative process of the neonate heart is characterized by clot formation at the site of injury coupled with an inflammatory response followed by epicardial cell and cardiomyocyte proliferation, ultimately leading to a restoration of cardiac function ^119, 120^. It is of interest that this regenerative capacity is lost during the first few days after birth ^119, 121^, which, as noted above, corresponds to the time point during which the terminal differentiation of cardiomyocytes is concluded ^109, 111^. Although these studies support the proposal that the mammalian neonatal heart possesses pronounced regenerative capacity, including the ability to replace cardiomyocytes, these results have been challenged by using the same experimental model in which limited cardiomyocyte proliferation and deficient neo-angiogenesis coupled with extensive scarring were observed ^122, 123^. These different outcomes have been suggested to be due to technical variation ^123– 126^, difficulties in tracking mouse cardiomyocyte proliferation during the first week of life ^127^, and timing of post-injury follow-up, where longer post-injury period allows more time for scar tissue deposition ^128^. Although there remains considerable debate regarding the efficiency of neonatal heart regeneration, it is generally agreed that the neonatal myocardium has some proliferative and angiogenic capacity which is lost in the adult heart ^128^. What might explain this more robust repair capacity during the first week of postnatal life in the mouse? As outlined in the previous section, the murine heart undergoes a metabolic switch to oxidative metabolism coupled with the maturation and bi-nucleation of cardiomyocytes, and the maturation of the coronary vasculature, which is driven by the availability of oxygen. This raises the hypothesis that low levels of oxygen during early postnatal life present a permissive context in which regeneration can occur.

## Tissue repair in the adult heart: adult cardiac progenitor cells

The adult mammalian heart is one of the least-regenerative organs in the body; thus, injury, most notably MI, leads to a progressive decrease in heart function and ultimately results in heart failure. In the past two decades, several studies have shown that the uninjured adult heart replaces a low percentage of the total cardiomyocytes ^129– 131^. Renewal of pre-existing cardiomyocytes is approximately 0.5% to 1% per year, a rate that declines with age ^129^. This low turnover rate implies that the vast majority of cardiomyocytes present at the conclusion of postnatal development remain throughout the entirety of adult life ^129, 132^. It has been demonstrated that adult cardiomyocytes undergo a fourfold increase in turnover following injury ^129, 132– 135^; however, the origin of new cardiomyocytes remains unclear. Several studies have provided evidence for the existence of adult resident CPCs that are able to give rise to cardiomyocytes and non-myocytes that represent a potential source for heart regeneration ( Table 1). CPCs have been defined and isolated by the expression of different markers clustered in niches in specific regions such as the atria, apex, and epicardium ^58, 136, 137^ ( Table 1); however, it is still unclear whether these different subsets belong to the same cell population or represent different CPC populations or do both. Additionally, the developmental origin of adult CPCs remains largely unknown, except for some of the CPC subsets that maintain a protein signature highly related with the embryonic populations such as the PEO-derived ^138– 140^ and Isl1+ ^141– 143^ subsets ( Table 1). After injury, CPCs are activated and give rise to different cell types (that is, myofibroblasts and smooth muscle cells and, to a lesser extent, endothelial cells and cardiomyocytes) ^144^. Of note, the contribution of these cells to new cardiomyocytes during steady-state and injury is highly debated ^145^. One high-profile example is the c-kit expressing CPCs, which have been reported to possess proliferative capacity and the ability to differentiate into cardiomyocytes ^146– 149, 171^, whereas other studies have refuted these observations and observe little to no cardiomyogenic potential ^172– 175^. Other studies have provided evidence demonstrating that the formation of new cardiomyocytes comes from pre-existing cardiomyocytes that proliferate in human steady-state young heart as well as in pathological conditions ^129, 135, 176– 178^. Similarly, in the mouse, studies have suggested that new cardiomyocytes are derived from pre-existing cardiomyocytes that re-enter the cell cycle ^120, 135, 172, 179– 182^ or arise from dedifferentiated myocytes ^183, 184^. Regardless of whether one or both of these models are accurate, it is clear that the adult heart is unable to functionally restore the adult myocardium following cardiac injury ^129, 135, 145, 185, 186^. In addition to the inefficient renewal of cardiomyocytes after injury, little revascularization is observed in the adult heart following injury, which likely contributes to poor cardiac regeneration ^187, 188^. Some studies revealed that endocardial cells, resident endothelial cells, or even fibroblasts can adopt an endothelial cell-like fate and contribute to angiogenesis in response to cardiac injury; however, the involvement of CPCs in this process remains largely controversial ^189– 191^. In the next section, we consider whether the potential of the heart to neovascularize following injury is a key process for future therapeutic targeting for heart disease.

**Table 1.** Different identified adult cardiac progenitor cell populations.

Adult cardiac progenitor cells	Embryonic origin	Progenies	References
**Identification/isolation by cell surface markers**			
c-kit+	Unknown	Cardiomyocytes, endothelial cells, and smooth muscle cells	146– 149, 150
Sca-1+	Unknown	Cardiomyocytes, endothelial cells, smooth muscle cells, and fibroblasts	151– 156
Islet-1+	SHF proposed	Smooth muscle cells, endothelial cells, parasympathetic neurons, sinoatrial node cells, and cardiomyocytes	20, 141– 143, 157, 158
Platelet-derived growth factor receptor alpha+ (PDGFRα+)	Unknown	Smooth muscle cells and endothelial cells	159, 160
Mesoangioblasts	Unknown	Cardiomyocytes	161, 162
Epicardial-derived progenitor cells	PEO	Coronary smooth muscle cells and adventitial and interstitial fibroblasts	56, 138, 140, 163
**Identification by dye-efflux function**			
Side population	Unknown	Cardiomyocytes, endothelial cells, smooth muscle cells, and fibroblasts	151, 164– 167
**Isolation by *in vitro* assays**			
Cardiospheres and cardiosphere-derived cells	Unknown	Cardiomyocytes	168– 170
Cardiac-resident colony-forming units-fibroblasts (cCFU-Fs)	PEO proposed	Fibroblasts	139

## The role of the adult epicardium in cardiac repair

**Figure 3.**
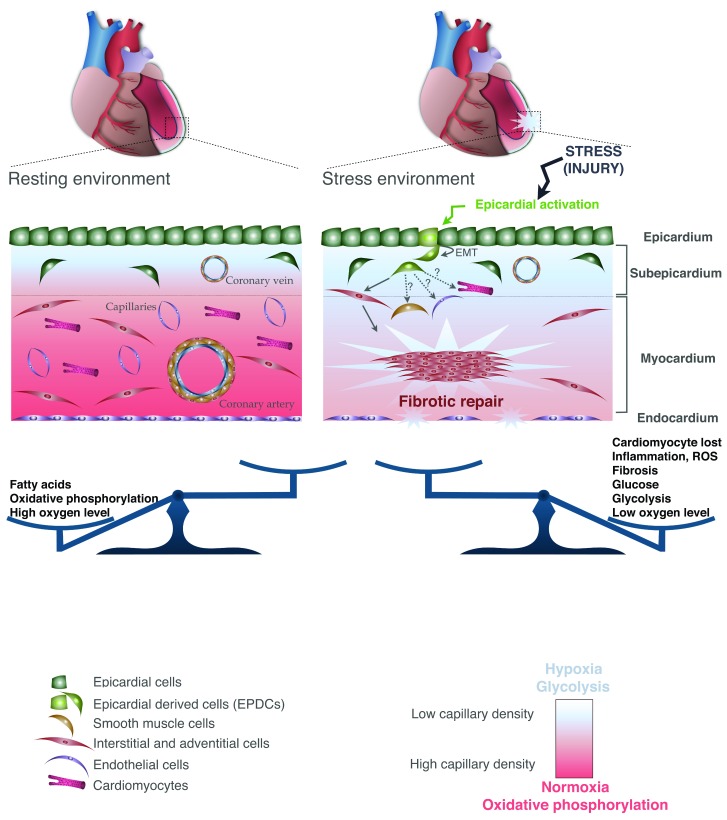
Adult heart under physiological conditions and after injury. Both the microenvironment and the cellular component are represented. A color code was assigned and followed to define each of the cardiac cell types and their associated cells (depicted in the legend). EMT, epithelial-to-mesenchymal transition.

The adult myocardium is surrounded and protected by the epicardium, which consists of a single mesothelial cell layer. As described in the previous section, the fetal epicardium is a source of cellular components and paracrine signals that promote coronary vasculature formation and myocardial growth ^192– 196^. Once the heart is mature, EPDCs progressively lose their capacity to undergo EMT and the epicardium enters a primarily quiescent state ^195, 197, 198^ ( Figure 3). In the adult, the role of the epicardium is to provide a physical barrier against infection ^199^. More recently, it has been shown that the epicardium is a key responder to heart injury ^195, 198, 200^ ( Figure 3). Specifically, the epicardium re-expresses genes typically observed during fetal epicardial development in response to injury, including *Wt1*, *Tbx18*, and *Raldh2*, coupled with proliferation and EMT to form a thick layer of EPDC mesenchymal cells ^163, 195^. EPDCs contribute to adventitial and interstitial fibroblasts and smooth muscle cells ^195, 201, 202^ ( Figure 3), suggesting that the adult epicardium serves as a reservoir for mesenchymal progenitor cells ^203^.

It has been shown that thymosin β-4 (Tβ-4) treatment results in adult epicardial activation, migration, and differentiation of EPDCs into smooth muscle cells, endothelial cells, and fibroblasts ^58, 204^. This is of interest since Tβ-4 is a key regulator of angiogenesis that acts through the stimulation of *Vegf* and stabilizing the hypoxia-inducible factor 1α ( *Hif-1α*) ^205^. It has been shown that Tβ-4 is expressed by the developing myocardium and regulates coronary vessel development (vasculogenesis, angiogenesis, and arteriogenesis) through activation of the epicardium ^58, 205^. Conversely, Banerjee and colleagues ^206^ could not confirm these results and claimed there is no link between Tβ-4 and angiogenesis during heart morphogenesis. The different transgenic models used in the two studies may explain the discrepancy of the results obtained. Tβ-4 is also able to promote the vascular potential of adult EPDCs, which contributes to an enhanced cardiomyocyte survival after injury ^58, 163, 205, 207^. Irrespective of the angiogenic properties of Tβ-4 during development, additional studies emphasize the involvement of epicardial cells and EPDCs in vasculature formation after cardiac injury. Wagner and colleagues ^208^ demonstrated that *Wt1*, the epicardial master transcription factor, is expressed in the coronary vessels of injured heart, suggesting a role for *Wt1* in the vascular growth after cardiac injury. Additionally, proangiogenic factors, such as VEGF and FGF, are secreted by EPDCs during the cardiac repair process, promoting the growth and survival of the heart vasculature ^195^.

In addition to providing a protective role for the heart, the epicardium serves as a reservoir for CPCs that are activated in response to injury. As such, the adult epicardium merits future research for targeting and ameliorating mammalian heart repair.

## The adult cardiac cellular microenvironment

The heart continually adapts to changing workloads brought about by aging, physical activities, and disease. The heart produces energy using multiple metabolic substrates such as fatty acids, glucose, ketone bodies, lactate, and amino acids ^209– 213^. It is estimated that the heart uses between 3.5 and 5 kg of ATP every day ^210^. Fatty acids are more energy-dense and thus provide more ATP molecules per consumed carbon as compared with the other substrates; however, fatty acid pathway requires more oxygen ^210, 211, 213^. A healthy heart uses primarily long-chain fatty acids, which provide 60% to 70% of the produced ATP to power the muscle contraction, while the remaining energy is derived from carbohydrate metabolism ^209– 211, 213, 214^. To meet this high ATP demand, the heart requires oxygen. When the body is at rest, myocardial oxygen consumption is greater than the oxygen consumption of any other organ of the body ^210, 211, 215^. Coronary circulation ensures the delivery of metabolic substrates and oxygen to the myocardium. Under physiological conditions, 90% of the ATP is produced through mitochondrial oxidative phosphorylation (fatty acids and glucose), which is an oxygen-dependent process ^216^. However, hypoxic conditions resulting from either physiological states (for example, during exercise) or pathological states (for example, coronary artery disease with mild ischemia) require the glycolytic pathway in order to produce ATP ^210, 211, 217^.

Although the adult heart is highly reliant on the availability of high levels of oxygen, the ventricles have a non-uniform distribution of oxygen across the wall with an oxygen tension gradient: lower tension in the extremities—that is, subendocardial (pO _2_ = 10 mmHg) and subepicardial (pO _2_ = 18 mmHg) regions—and higher tension in the middle of the myocardium (pO _2_ = 38 mmHg) ^218^. Reflecting the different levels of oxygen tension in the heart, Hif-1α, known to be stable under hypoxic conditions, is detected at high levels in the epicardium and subepicardium ^219, 220^. Although the high levels of environmental oxygen and adult stem cell niches (for example, bone marrow ^221– 223^) are characterized by low concentration of oxygen tension, this favors glycolytic metabolism. Although it has been shown to be essential, the exact mechanism of glycolytic metabolism and hypoxia in the maintenance of stemness properties is not well understood ^219, 223^. A protective mechanism of the adult stem cells against reactive oxygen species (ROS) has been implicit, but more studies are needed ^180, 181, 219^. As described previously, the epicardium represents a reservoir of adult CPCs and, like other adult stem cell niches, displays a low oxygen tension and relies on glycolysis-dependent metabolic pathways (cytoplasmic glycolysis) ^219, 224– 226^ ( Figure 3).

## Potential advantages of environmental manipulation to stimulate epicardial cells to a more efficient repair

Cardiovascular diseases are a major cause of human mortality ^227^. At present, long-term and effective treatments are missing. It is noteworthy that in high-altitude regions, cardiovascular diseases are less prevalent ^228– 230^, which has been attributed to continuous low-level hypoxia ^103, 231, 232^. Hypoxia activates an evolutionarily conserved adaptive process that allows mammals to cope with restricted oxygen tension. Indeed, as already described in this review, the hypoxic environment promotes a metabolic switch from aerobic mitochondrial metabolism to anaerobic cytoplasmic glycolysis, which is an essential feature of heart development driving cell proliferation, self-renewal, and differentiation ^78, 80– 83^ as well as of specific cardiac processes such as ventricular wall expansion through cardiomyocyte hyperplasia ^28, 30, 83^ or stimulation of angiogenesis and formation of coronary vasculature ^84– 87^. This feature of adult progenitor cells allows ATP production in the absence of oxygen, providing an advantage in a hypoxic environment ^219^. Additionally, it has become clearer that while oxidative metabolism is more efficient for producing ATP, cellular senescence and cell cycle arrest are a consequence of the resultant oxidative stress ^180^.

Although this review is focused on mammalian heart biology, it is important to note that lower vertebrates such as the zebrafish maintain a capacity to fully regenerate large domains of damaged myocardium through the proliferation of pre-existing cardiomyocytes ^233, 234^, which is similarly triggered by a hypoxic protective response ^235^. These results prompted Sadek and colleagues ^236, 237^ to explore the response of the mammalian heart to injury under hypoxic environmental conditions. Myocardial infarcted mice exposed for two weeks to hypoxia (7% oxygen) displayed a reduction of myocardial fibrosis and improved ventricular function. Sadek and colleagues proposed that the gradual reduction of environmental oxygen promotes glycolytic metabolism and reduces oxidative phosphorylation. This metabolic switch decreases ROS and cellular senescence and promotes the activation of proliferation of a small fraction of pre-existing cardiomyocytes ^236, 237^; however, it is also likely that additional progenitor populations contribute to this response. Additionally, Sadek and colleagues noted that there is a marked cardiac vascular network expansion (that is, the increase of coronary collaterals and capillary size) in the infarcted area after exposure to low levels of oxygen as compared with controls ^236^. As discussed above, the angiogenesis is dependent upon the oxygen tension and thus on the metabolic status of the microenvironment.

Emerging evidence suggests that the epicardium is an important source of stromal progenitor cells during development and that it maintains this capacity in the adult, potentially because of the particular hypoxic and glycolytic environment preserved in this adult heart compartment. While chronic exposure to high altitude carries collateral health risks in humans, it is tempting to propose that low oxygen tension can maintain the epicardium in an activated state, thus preserving perinatal plasticity, which in turn leads to improved cardiac repair. Further investigation of the adult CPCs and their microenvironment will provide important leads for improving cardiac repair following injury.

Taken altogether, while robust heart regeneration in the adult has yet to be achieved through therapeutic intervention, an understanding of the environmental clues and metabolic state of the developing heart will provide essential clues for novel therapeutic approaches. In the short term, it may be important to reconsider methodologies used during patient recovery following heart injury or insufficiency or both. Specifically, a more efficient angiogenic repair coupled with reduced scar tissue formation may result from promoting a more “embryonic-like” cardiac tissue environment during the repair process. How this can be achieved in a clinical setting and what additional clues can be translated to the clinic will be an interesting focus for the next decade.

## Authors’ contributions

AS and MV contributed to original draft preparation and to reviewing and editing. DS contributed to conceptualization and to reviewing and editing.
